# Enhancing the Startup Rate of Microbial Methanogenic Systems through the Synergy of β-lactam Antibiotics and Electrolytic Cells

**DOI:** 10.3390/microorganisms12040734

**Published:** 2024-04-03

**Authors:** Yuting Zhe, Huaigang Cheng, Fangqin Cheng, Huiping Song, Zihe Pan

**Affiliations:** 1 Institute of Resources and Environmental Engineering, Engineering Research Center of CO2 Emission Reduction and Resource Utilization—Ministry of Education of the People’s Republic of China, Shanxi University, Taiyuan 030006, China; zheyuting@foxmail.com (Y.Z.);; 2 College of Chemical Engineering, Qinghai University, Xining 810016, China

**Keywords:** microbial methanogenic system, inhibiting miscellaneous bacteria, rapid start technology, microbial community, PICRUSt2

## Abstract

The slow startup and suboptimal efficiency of microbial carbon sequestration and methane-production systems have not been fully resolved despite their contribution to sustainable energy production and the reduction of greenhouse gas emissions. These systems often grapple with persistent hurdles, including interference from miscellaneous bacteria and the slow enrichment of methanogens. To address these issues, this paper examines the synergistic effect of coupling β-lactam antibiotics with an electrolytic cell on the methanogenic process. The results indicated that β-lactam antibiotics exhibited inhibitory effects on *Campylobacteria* and *Alphaproteobacteria* (two types of miscellaneous bacteria), reducing their relative abundance by 53.03% and 87.78%, respectively. Nevertheless, it also resulted in a decrease in hydrogenogens and hindered the CO_2_ reduction pathway. When coupled with an electrolytic cell, sufficient electrons were supplied for CO_2_ reduction to compensate for the hydrogen deficiency, effectively mitigating the side effects of antibiotics. Consequently, a substantial improvement in methane production was observed, reaching 0.57 mL·L^−1^·d^−1^, exemplifying a remarkable 6.3-fold increase over the control group. This discovery reinforces the efficiency of methanogen enrichment and enhances methane-production levels.

## 1. Introduction

CO_2_ has considerable potential for reuse [1] and can be bioconverted by methanogens into methane through the digestion of organic waste [2], and microbial methanogenic systems utilize such archaea primarily for CO_2_ reduction and methane production. Nevertheless, the startup period of methanogenic microbial reactors is prolonged, ranging from 12 [3] to approximately 30 days [4], and the methanogen population often remains at low levels [5], especially when anaerobic digestion sludge is used as the inoculum. Consequently, this leads to a decrease in production efficiency [6]. The primary technical challenge stems from the presence of numerous other types of bacteria in the fermentation system throughout the microbial methane-production process. These miscellaneous bacteria coexist in substantial numbers and impose a substantial inhibitory influence on the methane-production procedure through competitive and antagonistic interactions [7].

To address the challenges stemming from the extended startup phase and the prevalent miscellaneous bacterial populations in methanogenic microbial reactors, a range of approaches have been employed. These include the manipulation of environmental conditions [8], the use of electrochemical methods [9], the addition of exogenous substances [10], and various other strategies. However, these tactics have not yielded a definitive resolution to the issue of interference from miscellaneous bacteria. Methanogens display a complex temperature dependency, with distinct optimal temperatures for the enrichment process and the methane-production process [8]. Additionally, altering pH has a substantial effect on methane production [11]. However, adverse environmental conditions can inhibit the activity of methanogens, and optimal environmental factors can only ensure that normal methane-production performance is obtained [12,13]. Besides environmental control, certain exogenous additives have been discovered to facilitate the enrichment of methanogens, such as algal biochar [10], cysteine [14], iron oxide [15], N-hexanoyl-L-homoserine lactone [16], and so on. However, these additives are often costly and challenging to obtain in large quantities. Furthermore, certain methods that enhance the startup phase of methanogens, including ultrasound [17], microwave [18], and biological enhancement [19], are operationally intricate or present challenges when it comes to scalability for industrial applications. The existing methods are partly constrained by the fact that they have not been extensively explored from the perspective of inhibiting interference from miscellaneous bacteria. The use of an electrolytic cell during the startup phase has been shown to have a positive impact. For the enrichment of high-performance methanogenic biofilms, it is important to guarantee a sufficient provision of reduction equivalents [20,21]. The application of an electric field can provide sufficient reducing equivalents and promote the enrichment of hydrotrophic methanogens [22]. Despite the demonstrated effectiveness of the electrolytic cell in the enrichment of methanogens, exploration into reducing interference from miscellaneous bacteria is still lacking. Consequently, it remains a significant and unresolved challenge in the realm of microbial methanogenesis to accurately identify the various types of miscellaneous bacteria within the methanogenic microbial system and explore strategies for restraining their growth and alleviating their detrimental effects on methanogenic communities.

On the other hand, an efficacious strategy to curb the proliferation of miscellaneous bacteria entails the deployment of antibiotics. Antibiotics are used to inhibit bacterial growth inhibition in the fields of medicine, food production, agriculture, and industry. They can alter the microbial community structure, leading to varying effects depending on the type, dosage, and duration [23]. Environmentally friendly antifungal antibiotics such as Tetramycin [24] and Imbricin [25] are employed within the realms of agriculture and the food industry. Moreover, antibiotics play an important role in preventing mold growth in industrial products [26]. In the context of methanogenic microbial systems, researchers have integrated antibiotic biodegradation processes into their investigation. However, it has been demonstrated that the majority of antibiotics exert inhibitory effects on methane production [27]. This could be attributed to the choice of antibiotic type and dosage. In this context, β-lactam antibiotics hold promise for inhibiting non-methane-producing bacteria, as they have been reported to have non-inhibitory effects on methanogens [28]. The mechanism entails a competition between β-lactam antibiotics and D-amino acids for the active site of the transport protein (PBP), resulting in damage to bacterial cell walls. Given that methane-producing archaea lack D-amino acids, β-lactam antibiotics effectively target bacteria without inhibiting methane-producing archaea. It is worth mentioning that there have been many studies showing that microbial reactors can enhance the breakdown of antibiotics in aquatic environments, and the degradation rate of amoxicillin can reach more than 90% [29,30], which makes the negative impact on the environment caused by the addition of antibiotics will be greatly reduced or even eliminated. Consequently, a thought-provoking question arises regarding its ability to address the issue of interference from miscellaneous bacteria within the methanogenic microbial system based on the fact that it can inhibit miscellaneous bacteria without inhibiting methanogens. Most other antibiotics would affect the methanogens. Should this methodology be validated for efficacy, and what might be the potential side effects of antibiotics on methane inhibition? Will the combination of antibiotics and an electrolytic cell consistently yield positive outcomes? To address these questions, it is essential to investigate the fundamental scientific aspects related to community succession in methane production and the regulatory mechanisms governing methane-production pathways when β-lactam antibiotics and electrolysis are coupled.

Taking into account the considerations outlined above, this study is pioneering in the use of a combination of β-lactam antibiotics and an electrolytic cell within a methanogenic microbial system. Through the examination of non-methanogenic miscellaneous bacteria and an exploration of community succession and regulatory mechanisms affecting methane-production pathways in this coupled system, this study aims to improve the startup rate of the methanogenic microbial system and provide a theoretical basis for the field of sustainable methane production.

## 2. Materials and Methods

### 2.1. Substrate and Inoculum

The anaerobic sludge employed in this research was sourced from the anaerobic fermentation tank at Shanxi Yuanping Muyuan Agriculture and Animal Limited Company, located in Xinzhou City, Shanxi Province, China. This inoculum was not the digestate from a full-scale digester, which has a lower abundance of methanogens and a higher abundance of miscellaneous bacteria. Initially, the collected anaerobic sludge underwent settling, and the supernatant was removed. Subsequently, it was passed through a 60-mesh sieve to eliminate larger particles. The filtered liquid underwent another settling process, with the resulting supernatant being discarded. The settled sludge was then sealed and stored in a refrigerator at 4 °C for future use. The methanogen culture medium constituted the growth substrate with the following specific formulation: K_2_HPO_4_ (0.4 g·L^−1^), KH_2_PO_4_ (0.4 g·L^−1^), MgCl_2_ (1.0 g·L^−1^), NH_4_Cl (1.0 g·L^−1^), 0.1% resazurin (2.0 mL·L^−1^), cysteine (0.5 g·L^−1^), Na_2_S (0.5 g·L^−1^), NaHCO_3_ (3.0 g·L^−1^), trace element solution (1.0 mL·L^−1^), and vitamin solution (1.0 mL·L^−1^).

### 2.2. Inoculation and Operation of Bioreactors

**Figure 1:**
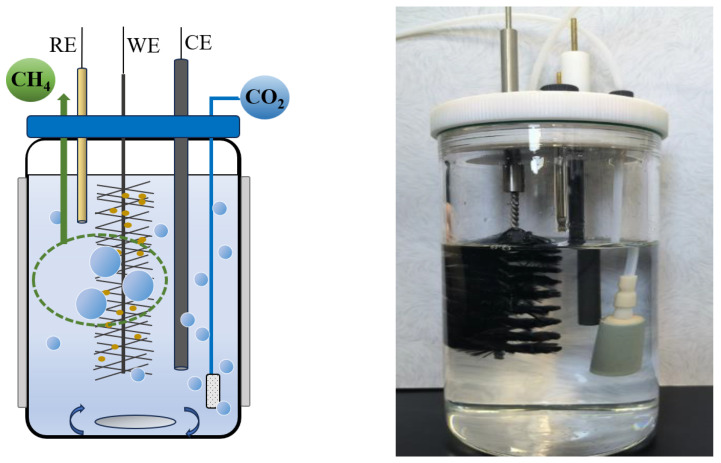
Schematic diagram of reactor unit.

The laboratory-scale bioreactor used in these experiments has dimensions of 15 cm in height, 7 cm in inner diameter, and a working volume of 400 mL. During inoculation, the culture medium was subjected to deoxygenation under elevated temperatures followed by a cooling phase to ambient conditions. It was then mixed with anaerobic sludge in a 3:1 volume ratio and promptly purged with N_2_ for 30 min to maintain an anaerobic environment. Pure CO_2_ was continuously supplied as the carbon source. Characteristics of the inoculums and culture medium are shown in Table 1. Throughout the operational period, the reactor solution composition remained constant, and a magnetic stirrer was employed to ensure the thorough mixing of the inoculum and substrate, thereby achieving a uniform dispersion of CO_2_ in the reactor. The temperature inside the reactor was maintained at 30 ± 1 °C using a thermal jacket. The reactor was equipped with a headspace for methane collection. The produced gas was collected using a syringe and analyzed for its composition and concentration using a gas chromatograph equipped with a Thermal Conductivity Detector (TCD) (SP-7890Plus, Lunan Rui Hong Chemical Instrument Co., Tengzhou, China). Each reactor was fitted with three electrodes: a working electrode in the form of a carbon brush (6 cm long, 3 cm in diameter), an auxiliary electrode represented by a graphite rod (10 cm long, 0.6 cm in diameter), and a reference electrode consisting of Ag/AgCl. A schematic diagram of the reactor unit is shown in Figure 1.

This study involved a group of reactors that employed sodium acetate as the carbon source. This group, referred to as the NaAc group, was supplemented with 5 g L^−1^ of CH_3_COONa in the culture medium. Notably, no antibiotics (ABX) were employed, and the three electrodes remained disconnected from a power source. Additionally, there were four groups of reactors that employed CO_2_ as the substrate. These groups included the ABX.E group, which combined amoxicillin and an electrolytic cell with an amoxicillin concentration of 1 × 10^5^ U L^−1^ (the effects of different types and concentrations of β-lactam antibiotics were investigated in a previous pre-test, and the results showed that 1 × 10^5^ U/L of AMX was the most effective), and the operation was sustained for a prolonged duration under an applied voltage of 0.6 V, utilizing an electrochemical workstation for voltage control; the ABX group, using only amoxicillin with a concentration of 1 × 10^5^ U L^−1^; the E group, operating solely at an applied voltage of 0.6 V; and the CG, where neither amoxicillin nor an electrolytic cell was employed. The anaerobic environment required for the growth of methanogens was maintained throughout all the reactors in this research. The experimental grouping and reactor setup are shown in Table 2. The reactor solutions were sampled at 7-day intervals for measurement, with the entire experimental period lasting 35 days and encompassing a total of five stages. All experiments were repeated three times to ensure reliable data.

### 2.3. Illumina Sequencing and PICRUSt2 Analysis

High-throughput sequencing was conducted on the Illumina MiSeq platform by Novogene Co., Ltd., based in Beijing, China. For PICRUSt2 analysis, DNA extraction and PCR amplification were first required to obtain raw data. The raw data potentially encompassed a subset of low-quality data. To ensure the accuracy and reliability of subsequent data analysis, the raw data underwent initial processing involving trimming and filtering to yield clean data. Subsequently, denoising was carried out on the clean data using DADA2 or deblur (with the default choice being DADA2), resulting in the generation of the final Amplicon Sequence Variants (ASVs). Following the acquisition of ASVs, species annotation was applied to the representative sequences of each ASV, facilitating the retrieval of species-related information and the distribution of species abundances. Additionally, an array of analyses was carried out on the ASVs, encompassing assessments of abundance, alpha diversity, the generation of Venn diagrams, and the creation of heatmaps. These analyses offer insights into species richness and evenness within the samples, as well as the identification of shared and unique ASVs across different samples or groups. Furthermore, multiple sequence alignment was employed on the ASVs to construct a phylogenetic tree. Metabolic functions were predicted using PICRUSt2 software (version number: PICRUSt2 V2.3.0), and the anticipated gene abundances encoding enzymes within the bacterial community were investigated with respect to MetaCyc pathways.

## 3. Results

### 3.1. Evolution in the Composition and Structure of Methane-Producing Community

The evolution of methane-producing community composition plays a pivotal role in influencing methane-production performance. This process raises two critical issues: firstly, it seeks to identify non-methanogenic bacteria present within the methane-producing community; and secondly, it explores the potential for inhibiting these miscellaneous bacteria to facilitate the enrichment of methanogens.

**Figure 2:**
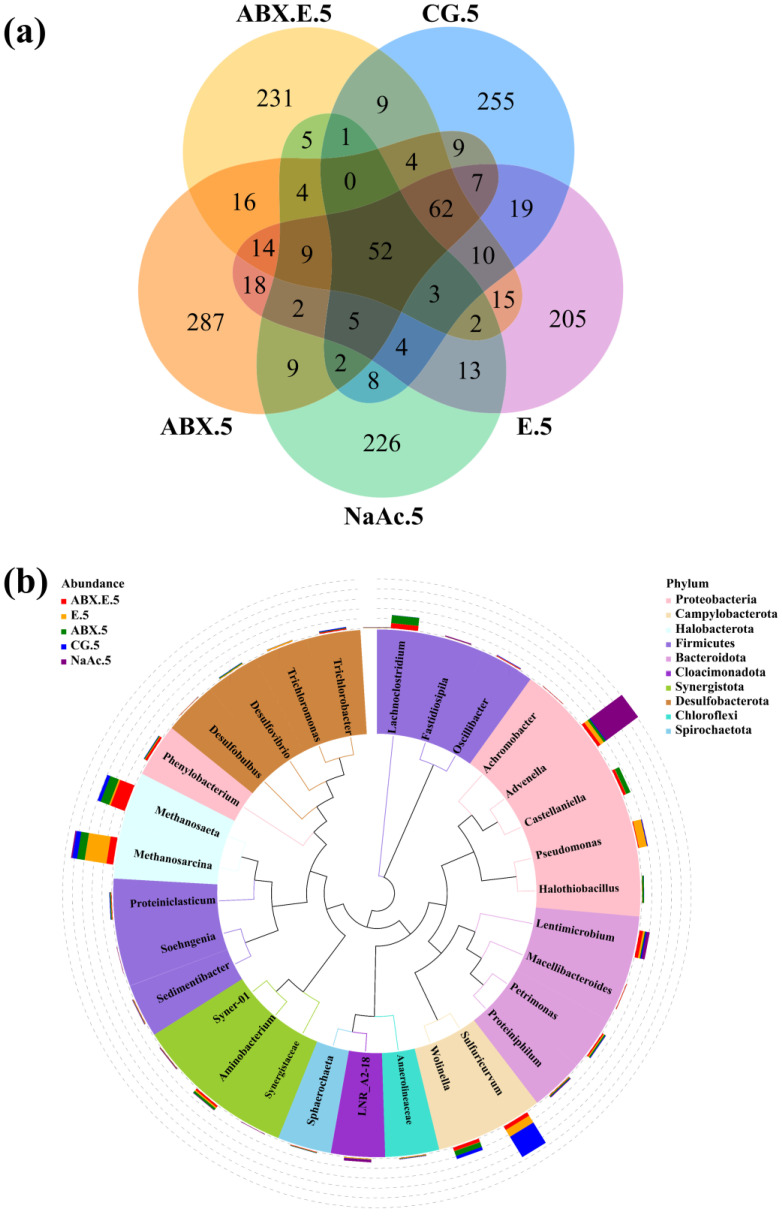
Structure of methane-producing community: (**a**) Number of total OTUs for ABX.E.5, E.5, ABX.5, CG.5, and NaAc.5; (**b**) Species evolutionary tree at genus level.

To identify the miscellaneous bacteria, distinctions in community dynamics and species composition were determined. The total and unique Operational Taxonomic Units (OTUs) at the final stage of community composition evolution under different experimental conditions can indicate variations in the evolution trend. Figure 2a illustrates the enumeration of OTUs corresponding to various experimental conditions at the culminating phase of the study. Among them, ABX.E.5, E.5, ABX.5, CG.5, and NaAc.5 collectively possess 52 shared OTUs, while the numbers of unique OTUs for these conditions are as follows: 231, 205, 287, 255, and 226, respectively. Significant differences in methane-producing community composition have become evident as a result of the influences of antibiotics and electrolysis. As depicted in Figure 2b, at the genus level, the predominant methanogens in this study are *Methanosarcinia* and *Methanosaeta*, with abundances of 13.3% and 9.1%, respectively. The bacteria with the highest abundances, ranking in the top thirty, mainly belong to the following ten classes: bacteria implicated in the methane generation process encompass *Gammaproteobacteria* (involved in lactose fermentation, yielding acid and gas), *Bacteroidia* (participating in carbohydrate fermentation and the consumption of nitrogenous substances, with the primary byproduct being acetic acid), *Clostridia* (serving as vital agents for nitrogen fixation and cellulose degradation, resulting in acetic acid production), *Synergistia* (reciprocal fermentation bacteria that often coexist with hydrogen-producing bacteria), *Desulfuromonadia*, and *Desulfovibrionia* (principally carrying acid fermentation genes). On the other hand, the miscellaneous bacteria not associated with methane production, which compete with methanogens for resources, predominantly comprise *Campylobacteria* and *Alphaproteobacteria*.

**Figure 3:**
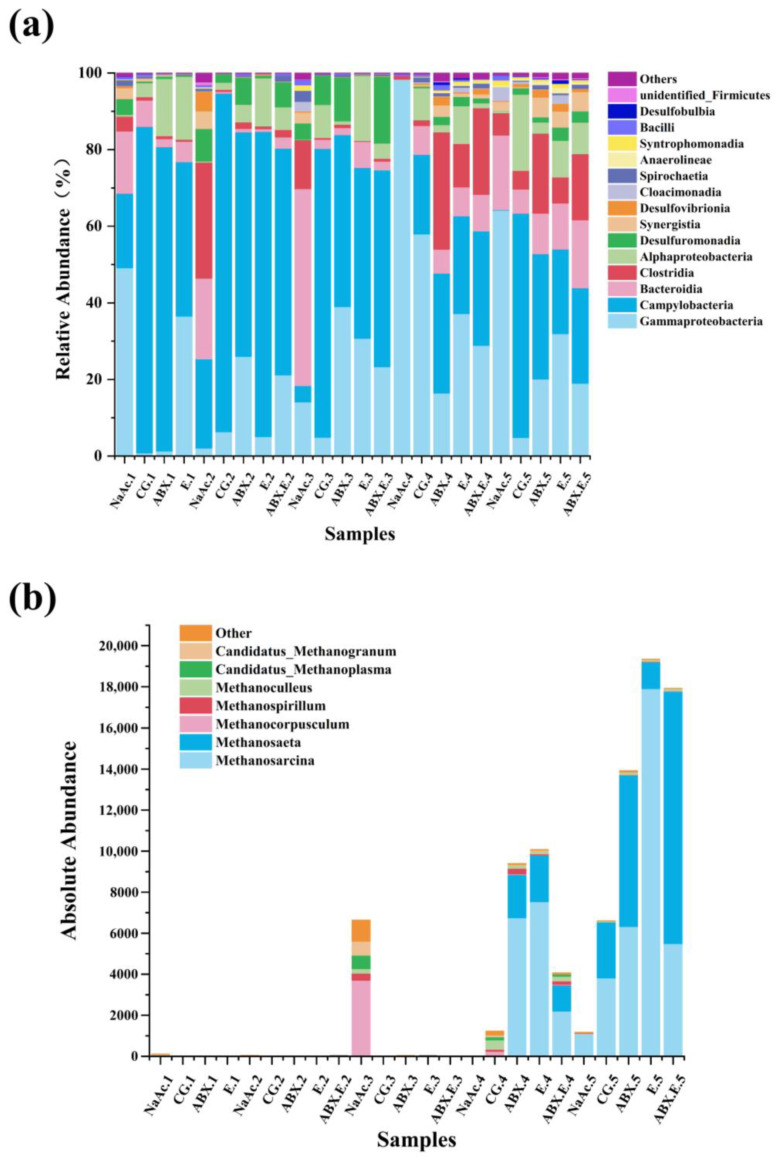
Evolution of methane-producing communities: (**a**) Histogram of stacked relative abundance percentages of bacteria; (**b**) Histogram of stacked absolute abundance of archaea.

To conduct a comprehensive analysis of the dynamic patterns of miscellaneous bacteria and the effects of antibiotics and electrolytic cells, the temporal variation in the relative abundance of the diverse bacterial community was evaluated. Figure 3a displays the changes in the relative abundance of bacteria. The primary miscellaneous bacteria, namely *Campylobacteria* and *Alphaproteobacteria*, are predominantly Gram-negative bacteria, and they do not contribute positively to methane production; instead, they compete with methanogens for resources. With increasing cultivation time, the relative abundance of *Campylobacteria* and *Alphaproteobacteria* shows a reduction. By the 35th day, a significant decrease in the relative abundance of *Campylobacteria* was observed in the ABX group (with antibiotics), E group (application of electrolytic cell), and ABX.E group (combining both), which decreased by 53.03%, 72.73%, and 68.00%, respectively, in comparison to the control group. Similarly, the relative abundance of *Alphaproteobacteria* in the ABX group, E group, and ABX.E group decreased by 87.78%, 65.41%, and 68.91% respectively, compared to the control group. This observation indicates that the primary miscellaneous bacteria demonstrate a decreasing trend under the influence of antibiotics. This decline can be attributed to the antibacterial activity of amoxicillin, which hinders the growth and reproduction of *Campylobacteria* and *Alphaproteobacteria* by interfering with bacterial cell wall synthesis [28]. It is noteworthy that β-lactam antibiotics also reduced the relative abundance of hydrogenogens. *Clostridiales_bacterium* could be detected in the first two stages in the ABX group and was undetectable in the fifth stage, but it could still be detected during the same period in the control group with no antibiotic added. The relative abundance of *Clostridium_butyricum* in the ABX group was reduced by 11.04% in comparison to the control group without antibiotic addition at the fifth stage. On the other hand, electrolytic cells tend to promote the growth and reproduction of methanogens [31]. Consequently, it is reasonable to speculate that the reduction in the relative abundance of miscellaneous bacteria in the E group is indirectly caused by the increase in methanogens.

In addition to assessing the alterations in miscellaneous bacteria, it is essential to analyze whether antibiotics and electrolytic cells have the potential to enhance the enrichment of methanogens. At the genus level, the predominant methanogens in this study are *Methanosarcina* and *Methanosaeta*, with a ratio of 1:0.57 across all samples. *Methanosarcina* primarily metabolizes H_2_/CO_2_ or acetate, while *Methanosaeta* exclusively utilizes acetate as a substrate and does not use H_2_/CO_2_. Figure 3b displays the relative trend of variation in methanogens, which demonstrates rapid proliferation commencing in the third week. By the fifth week, influenced by antibiotics and electrolysis, the relative abundance of methanogens in the ABX group, E group, and ABX.E group demonstrates significant increases compared to the CO_2_ control group, with increments of 109.60%, 193.37%, and 171.73%, respectively. Additionally, when compared to the NaAc control group, these experimental groups exhibited 12.26-fold-, 17.16-fold-, and 15.89-fold-higher relative abundances of methanogens, respectively. This observation suggests that at a concentration of 1 × 10^5^ U L^−1^, β-lactam antibiotics, represented by amoxicillin, indirectly stimulate the enrichment of methanogens by inhibiting miscellaneous bacteria. Additionally, the employment of an electrolytic cell at 0.6 V has been shown to substantially bolster the methanogenic population.

Furthermore, it is necessary to consider what competitive mechanism the CO_2_ injected into the reactor provides for competition between bacteria and archaea. Methanogens are usually in a symbiotic relationship with bacteria involved in anaerobic digestion and in a competitive relationship with other bacteria not involved in anaerobic digestion. Normally, because methanogens need bacterial metabolites as nutrients, they need to complete the three stages of hydrolysis, acidification, and hydrogen and acetic acid production before they can undergo rapid growth and reproduction. However, exogenous CO_2_ changes the relationship between populations, methanogens can utilize CO_2_ directly for methanogenesis, and no longer need the metabolites produced in the first three phases as a carbon source, in which case methanogens become competitive with all other bacteria except for hydrogenogens. It can be specifically inferred that the injection of exogenous CO_2_ exerts the following impacts on the microbial dynamics: (1) The exogenous CO_2_ brought an additional carbon source for the methanogens, which was more favorable to the growth of the methanogens. (2) At the same time, when the exogenous CO_2_ was injected, no other organic carbon source was added to the reaction system and most of the bacteria could not utilize the inorganic carbon source, and the remaining organic carbon source in the system was consumed over time, so the growth of such bacteria was suppressed to a certain extent, which indirectly enlarged the living space of the methanogens and favored the growth and proliferation of the methanogens. In summary, the injection of CO_2_ into the reactor inhibits the growth of bacteria to some extent while promoting the growth of methanogenic archaea.

The findings presented above suggest that the miscellaneous bacteria Campylobacteria and Alphaproteobacteria, which can be inhibited by amoxicillin, are suppressed in their relative abundance, allowing more space for the growth and reproduction of methanogens in the methanogenic microbial system. Consequently, this facilitates the rapid growth and proliferation of methanogens. Furthermore, the use of an electrolytic cell also contributes to the acceleration of methanogen enrichment within the system.

### 3.2. Methane-Production Performance

Methane-production performance serves as the ultimate reflection of the evolution of the methane-producing community. In this section, the viability of antibiotics and electrolysis, both individually and in combination, in this process is explored through an examination of methane-production measurements.

**Figure 4:**
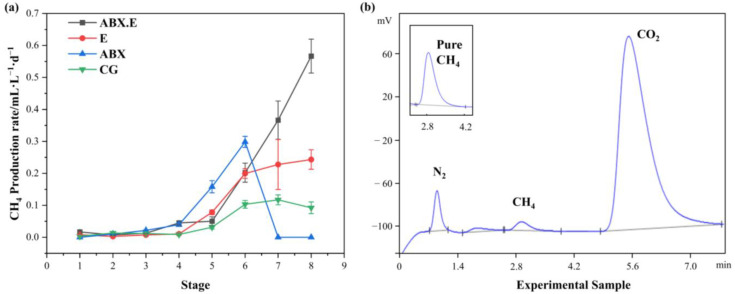
The methane-production rate under different experimental conditions: (**a**) Methane-production rate; (**b**) Gas chromatogram of experimental sample.

By conducting systematic assessments of methane production across varied experimental conditions, the changing trend of methane production over time is observed (Figure 4). On the whole, the methane-production rate gradually increases over time during the initial six stages. For the CG, there is a moderate increase followed by a subsequent decline, reaching a peak rate of 0.18 mL·L^−1^·d^−1^ in the seventh stage. This pattern can be attributed to the gradual enrichment of methanogens, leading to the initial rise in methane production. However, the ensuing decline appears incongruous with the trends witnessed within the microbial assemblies, necessitating further analysis, particularly with regard to metabolic pathways.

Moreover, the variations in methane production within the ABX group and the E group are also depicted in Figure 4a. Figure 4b shows the gas chromatogram of the outlet gas when the reactor was started, and a peak appeared at 2.8 min. Comparing it with the standard CH_4_ sample, it can be confirmed that this is a methane peak. The ABX group demonstrates the most rapid growth in methane production during the initial six stages, attaining a maximum rate of 0.30 mL·L^−1^·d^−1^. This acceleration is attributed to the rapid enrichment of methanogens during the growth phase. However, it experiences a sharp decline after the seventh stage. In contrast, the E group maintains a relatively high methane-production rate beyond the sixth stage and can sustain this heightened rate even in the eighth cycle, eventually stabilizing at a higher level of 0.24 mL·L^−1^·d^−1^. On the whole, the separate use of antibiotics and electrolytic cells can improve methane yield to some extent, although the enhancements are modest—only 2.08 to 2.55 times that of the CG.

Finally, observations were made regarding the changes in methane production in the ABX.E group. Following a gradual growth in the first five stages, the ABX.E group enters rapid growth. At phase five, the lower methane production in the ABX.E group compared to the E group may be due to experimental errors caused by operational or changes in environmental conditions. By the eighth stage, the methane-production rate reaches 0.57 mL·L^−1^·d^−1^, surpassing the second-ranked E group by 2.4 times and outperforming the CG group by 6.3 times within the same period. This indicates that the coupling of antibiotics and electrolytic cells can achieve the highest methane-production efficiency, in accordance with the findings of the methanogenic community evolution depicted in Figure 3. This augmented methane-production capability is a consequence of the microbial consortium’s adaptation and evolution.

In summary, methane yield during the growth phase generally follows the trend of community evolution. The collective impact of antibiotics and electrolytic cells on methane-production performance surpasses their individual effects, highlighting the feasibility of this approach. However, the observed decline in methane-production levels in the CG and ABX groups in the later stages, which contradicts the rising trend of the methane-producing community depicted in Figure 3b, necessitates further analysis, taking into account the metabolic pathways.

### 3.3. Function and Metabolic Pathway Analysis Based on PICRUSt2 Predictions

The decrease in methane-production levels in the later stages cannot be accounted for solely by community evolution. Therefore, a metabolic pathway analysis of methane-production processes was conducted.

**Figure 5:**
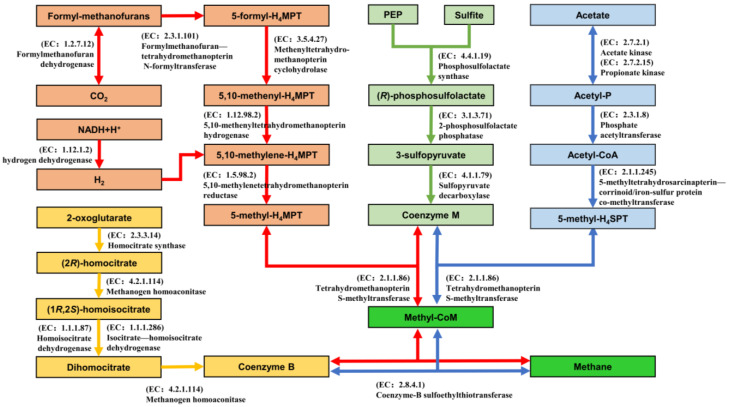
Biochemical pathways for methane production. (blue arrows: the acetate pathway; red arrows: the H_2_/CO_2_ pathway; green arrows: the pathways for the generation of Coenzyme M; yellow arrows: the pathways for the generation of Coenzyme B).

Initially, the biochemical metabolic pathways for microbial methane production were established using the MetaCyc database [32]. Subsequently, the metabolic pathways were analyzed using PICRUSt2 [33] to predict the abundance of genes associated with methane-production pathways. Within the methane-production metabolic pathway, the primary divisions include the acetate pathway and the H_2_/CO_2_ pathway. Figure 5 elucidates the acetate pathway [34], the H_2_/CO_2_ pathway [35], and the pathways for the generation of two main coenzymes, Coenzyme M [36] and Coenzyme B [37]. This exploration aims to shed light on the methane-production process under different conditions.

**Figure 6:**
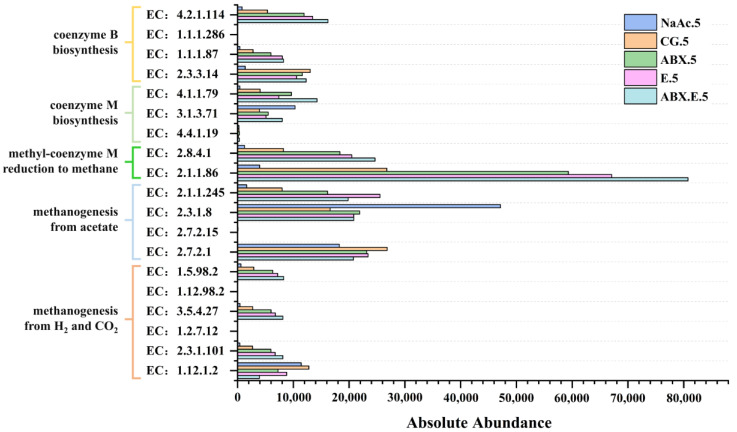
Abundance prediction of enzymes involved in biochemical pathways of methane production.

Furthermore, following the delineation of the metabolic pathways, it becomes imperative to assess the abundance of enzymes pertinent to each pathway. Figure 6 depicts the predicted abundance of enzymes associated with methane-production pathways. The trend of enzymes associated with methane-production pathways displays significant variations under different cultivation conditions. In the majority of enzymes, abundance is ranked as ABX.E group > E group > ABX group > CG, highlighting that the collective impact of antibiotics and electrolytic cells surpasses their individual effects. Within the ABX.E group, the abundance of enzymes associated with the H_2_/CO_2_ pathway formylmethanofuran—tetrahydromethanopterin N-formyltransferase (EC: 2.3.1.101), methenyltetrahydro-methanopterin cyclohydrolase (EC: 3.5.4.27), and 5,10-methylenetetrahydromethanopterin reductase (EC: 1.5.98.2) was greater than those of the other groups—3.0, 3.0 and 2.9 times that of the CG. Similarly, the abundance of enzymes related to coenzyme M formation, such as 2-phosphosulfolactate phosphatase (EC: 3.1.3.71) and sulfopyruvate decarboxylase (EC: 4.1.1.79); coenzyme B formation, such as methanogen homoaconitase (EC: 4.2.1.114) and homoisocitrate dehydrogenase (EC: 1.1.1.87); and enzymes associated with methyl-coenzyme M formation, such as tetrahydromethanopterin S-methyltransferase (EC: 2.1.1.86), and coenzyme-B sulfoethylthiotransferase (EC: 2.8.4.1), also exceeds that in the other groups—approximately 2.1~3.5 times that of the CG. Conversely, the enzymes related to the acetate pathway are at a lower level. This indicates that the ABX.E group predominantly utilizes the H_2_/CO_2_ pathway for methane generation in response to the synergistic application of antibiotics and electrolytic cells, and the overall methane-production efficiency is higher than the other groups.

The disparities in metabolic pathways are evident when electrolytic cells and antibiotics are applied individually or in combination. In the ABX group, with the sole use of antibiotics, the abundance of enzymes associated with the H_2_/CO_2_ pathway is the lowest among the experimental groups, ranging from 73.7% to 76.1% of the ABX.E group. Conversely, the abundance of enzymes related to the acetate pathway is higher, ranging from 81.5% to 111.5% of the ABX.E group. This deviation could be ascribed to the antibiotic-induced diminution in the relative abundance of hydrogenogens within the ABX group, which subsequently obstructs the H_2_/CO_2_ pathway. Specifically, the abundance of the key hydrogenase enzyme hydrogen dehydrogenase (EC: 1.12.1.2) in the ABX group was only 63.5% of that in the NaAc group and 56.8% of that in the CG; for the other group, the ABX.E group, with antibiotics added, the abundance of this EC was only 34.2% of the NaAc group and 30.6% of the CG. This indicates that ABX significantly inhibited the H_2_ production pathway and the H_2_/CO_2_ pathway, so the ABX group was dominated by the acetate pathway for methanogenesis. In the E group, there is a relatively high abundance of enzymes associated with both the acetate pathway and the H_2_/CO_2_ pathway. In fact, the abundance of coding genes associated with the H_2_/CO_2_ pathway is second only to the ABX.E group, ranging from 83.1% to 87.0% of the ABX.E group. This is due to the electrons provided by the electrolytic cell. Significantly, this group demonstrates the capacity to yield methane from not only sodium acetate but also via CO_2_ utilization.

Through function and metabolic pathway analysis, it was found that ABX can inhibit the synthesis of H_2_ and block the H_2_/CO_2_ pathway, thereby inducing the acetate pathway to dominate the methane-production process, resulting in poor performance when CO_2_ is used as the substrate for methane production. Electrolytic cells can provide sufficient reductive equivalents, replacing H_2_ to reduce CO_2_ to methane. When both ABX and electrolytic cells are used simultaneously, the electrons provided by the electrolytic cell compensate for the lack of H_2_, enhancing the H_2_/CO_2_ pathway. At the same time, because miscellaneous bacteria are suppressed, the abundance of methanogens increases, and the ABX.E group achieves the best methane-production performance. Figure 5 shows the biochemical pathways for methane production.

## 4. Discussion

This section will aim to elucidate the mechanisms by which β-lactam antibiotics and electrolytic cells function in microbial methanogenic systems by analyzing the experimental results. It will also discuss the positive impacts of this study on the biogas industry, CCUS (Carbon Capture, Utilization and Storage), and other fermentation industries, and propose future research directions in the field of microbial methane production.

**Figure 7:**
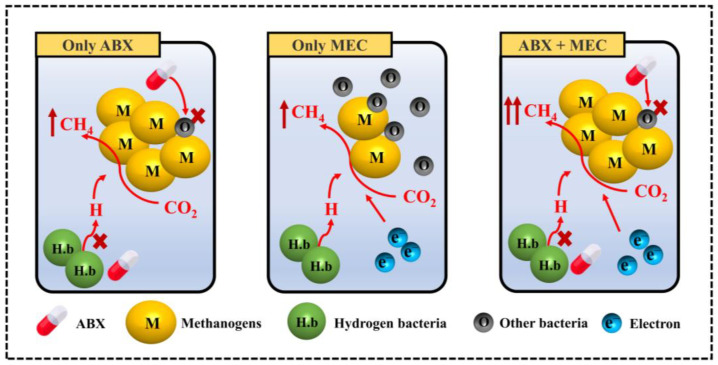
Illustration of methane-production process under different conditions.

The role of β-lactam antibiotics and electrolytic cells in microbial methanogenic systems is depicted in Figure 7. The β-lactam antibiotic represented by AMX can effectively inhibit the growth and reproduction of non-methanogenic miscellaneous bacteria. By weakening the competition and antagonism against methanogens from these miscellaneous bacteria, the growth and enrichment of methanogens are accelerated. However, ABX also inhibits the H_2_ production pathway, further hindering the H_2_/CO_2_ pathway, so methane production is dominated by the acetate pathway. Subsequent to the sixth stage, as shown in Figure 4, the ABX group experienced a pronounced reduction in methanogenic activity—a trend that could be ascribed to the exhaustion of sodium acetate. On the other hand, when only electrolytic cells are employed, the applied voltage is effective in promoting the enrichment of methanogens and increasing the methane-production rate. Both the acetate pathway and the H_2_/CO_2_ pathway are operational, enabling methane production from both acetate and CO_2_. In this scenario, the reducing equivalents can originate from hydrogen gas generated by hydrogenogens or from electrons supplied by the cathode. These factors contribute to the E group demonstrating superior methane-production performance, aligning with the observed methane-production rate.

In the coupling of β-lactam antibiotics and electrolytic cells, the ABX.E group predominantly utilized the H_2_/CO_2_ pathway for methane production and exhibited a higher methane-production rate than the other groups. The commonality with the ABX group without electrolytic cells is that the presence of antibiotics in both cases inhibits the growth of miscellaneous bacteria and hydrogenogens and promotes the growth and proliferation of methanogens. However, the distinguishing factor lies in the external voltage, facilitating the migration of electron mediators from the cathode to the cytoplasmic matrix of methanogens in the ABX.E group. This enables the following reaction between the electron and bicarbonate ions, as depicted in Equation (1). The introduction of electrons as a new source of reducing equivalents compensates for the shortage of hydrogen, enabling the ABX.E group to continue utilizing CO_2_ as a substrate for methane production. In comparison to the E group without antibiotics, both groups primarily rely on the reduction of CO_2_ for methane production. However, the presence of antibiotics in the ABX.E group diminishes the interference of non-methanogenic miscellaneous bacteria in the methane-production process, leading to enhanced methane-production performance. To summarize, while β-lactam antibiotics suppress various miscellaneous bacteria, they inadvertently impede the H_2_ production pathway. However, integration with electrolytic cells overcomes this hindrance, synergistically enhancing the overall methane-production efficacy.

HCO_3_^−^ + 9H^+^ + 8e^−^→CH_4_ + 3H_2_O (1)

The findings have positive implications for the biogas industry, CCUS, and other fermentation industries. To begin with, in the biogas industry, the enrichment and startup rate of methanogens directly determine production efficiency. Traditional methods involve a prolonged period of enrichment with significant interference from miscellaneous bacteria. This study has markedly improved the enrichment rate of methanogens and the rate of methane production by combining β-lactam antibiotics with electrolytic cells. This provides a novel and more efficient method for methane production, which could accelerate the industrialization process of biogas production and foster the production of sustainable energy. In the realm of CCUS, enhancing the efficiency of biogas production strengthens the transformation and utilization of CO_2_, aiding in the reduction of greenhouse gas emissions and addressing global climate change. Finally, within the fermentation industry, microbial metabolism and fermentation processes are often disrupted by miscellaneous bacteria. The inhibitory effect of β-lactam antibiotics on such miscellaneous bacteria found in this study could also provide insights into the fermentation industry. Moreover, the application of electrolytic cells can offer new sources of energy and reaction conditions for the fermentation industry, driving technological innovation and industrial upgrading. The findings of this study are anticipated to be broadly adopted and promoted in the future, contributing significantly to sustainable development and environmental protection.

In the research and application of microbial methanogenic systems, future research directions will revolve around several core issues: (1) Priority should be given to a deeper exploration of the symbiotic and competitive dynamics between methanogens and other bacteria. Understanding their competitive and cooperative relationships can help better control the growth of miscellaneous bacteria, increase the enrichment efficiency of methanogens, and boost methane yield. Furthermore, the breeding and genetic engineering of methanogens to boost their environmental resilience and methane output efficiency will establish a solid base for their engineering deployment. (2) The industrial application and commercialization of biogas are also priorities for future research. This includes the development of reactor systems suitable for large-scale production, optimizing production processes, reducing production costs, and increasing the purity of methane. It is also necessary to study how to integrate biogas more effectively into the current energy infrastructure to achieve its widespread application in the energy field. (3) Microbial methanogenic systems require high-purity CO_2_ source gases, including a concentration of CO_2_ in the gas, oxygen levels, and other harmful substances. However, industrial waste gases with high CO_2_ content often fail to meet the requirements. Researching how to obtain high-quality CO_2_ gases in production and achieving the efficient capture, oxygen removal, and purification of industrial waste gas CO_2_ is crucial to the industrial application of microbial methanogenic systems.

In summary, future research directions in the field of methanogens research and application will involve aspects such as microbial interactions, industrial application, and CO_2_ sources in practical production. Through in-depth research and continuous exploration, it is anticipated that the field will achieve more breakthroughs and progress, thereby making significant contributions to sustainable development and environmental protection.

## 5. Conclusions

In this work, the synergistic effect of β-lactam antibiotics in combination with an electrolytic cell on the methanogenesis process was investigated. The findings revealed that the β-lactam antibiotic amoxicillin indirectly inhibited miscellaneous bacteria but did not inhibit methanogens. This inhibition expanded the living space for methanogens and facilitated their enrichment. However, the antibiotic inhibition of miscellaneous bacteria and hydrogenates blocked the H_2_/CO_2_ pathway. Electrolytic cells can supply adequate electrons as new reducing equivalents, compensating for the shortage of hydrogen and restoring the blocked H_2_/CO_2_ pathway. This synergistic approach addresses the side effects of antibiotics, resulting in significantly increased methane-production performance. This study successfully addresses the challenge of a high abundance of non-methanogenic miscellaneous bacteria by combining antibiotics and electrolytic cells.

## Figures and Tables

**Table 1:** Characteristic of the inoculums and culture media.

Parameters	Inoculums	NaAc Culture Medium	CO_2_ Culture Medium
pH	7.5	7.0	7.0
TS ^1^ (mg/L)	130	-	-
VS ^2^ (mg/L)	70	-	-
TCOD ^3^ (mg/L)	4100	4410	510
SCOD ^4^ (mg/L)	2680	4410	510
TP ^5^ (mg/L)	138	165	162
TN ^6^ (mg/L)	2100	1550	1500
NH_4_^+^-N ^7^ (mg/L)	940	290	285

^1^ Total Solid; ^2^ Volatile Solid; ^3^ Total Chemical Oxygen Demand; ^4^ Soluble Chemical Oxygen Demand; ^5^ Total Phosphorus; ^6^ Total Nitrogen; ^7^ Ammonium Nitrogen.

**Table 2:** Experimental grouping and reactor setup.

Abbreviation	Carbon Source	Antibiotics	Electrolytic Cell
NaAc	CH_3_COONa	×	×
CG	CO_2_	×	×
ABX.E	CO_2_	√	√
ABX	CO_2_	√	×
E	CO_2_	×	√

## Data Availability

The sequencing data have been uploaded to NCBI, BioProject ID: PRJNA1056758.3.
